# Vicarious Stigma and Self-Stigma Experienced by Parents of Children with Mental Health and/or Neurodevelopmental Disorders

**DOI:** 10.1007/s10597-021-00774-0

**Published:** 2021-01-21

**Authors:** Marisa D. Serchuk, Patrick W. Corrigan, Sarah Reed, Jeneva L. Ohan

**Affiliations:** 1 Department of Psychology, Illinois Institute of Technology, 3424 South State Street, Chicago, IL 60616 USA; 2 Rogers Behavioral Health, 4555 W. Schroeder Drive, Suite 185, Brown Deer, WI 53223 USA; 3 School of Psychological Science, University of Western Australia, M304, Perth, WA 6009 Australia

**Keywords:** Stigma, Vicarious stigma, Self-stigma, Secrecy coping, Disclosure, Parents

## Abstract

The stigma of young children with mental health and/or neurodevelopmental disorders is experienced by their parents in at least two ways: self-stigma and vicarious stigma. Secrecy may diminish stigma through impression management or strategic disclosure. The present study explores the relationship between vicarious stigma, self-stigma, secrecy coping, depression, and quality of life. Additionally, we examine the structure of a novel measure of vicarious stigma. Fifty parents of children with mental health and/or neurodevelopmental disorders completed measures. Self-stigma and sadness due to vicarious stigma were significantly associated with greater depression and diminished quality of life. Higher secrecy coping was also associated with higher depression and lower quality of life, supporting the benefits of disclosure. This research meaningfully adds to our understanding of stigma in general, and as experienced by parents of children with mental health and/or neurodevelopmental disorders. Implications for ongoing stigma change development and evaluation are discussed.

## Introduction

Parents of children with mental health and/or neurodevelopmental disorders experience stigma. Researchers have distinguished at least two types of stigma in general: (a) public stigma, the prejudice and discrimination experienced by a labeled group when the population endorses stereotypes about that group, and (b) self-stigma (SS), harm to self-esteem and self-efficacy when someone internalizes these stereotypes (Brohan, Slade, Clement, & Thornicroft, 2010). Research clearly shows that even young children with mental health (e.g., depression) or neurodevelopmental [e.g., attention-deficit/hyperactivity disorder (ADHD), autism spectrum disorder] disorders are stigmatized by the general public with stereotypes including dangerousness and blame (Kinnear, Link, Ballan, & Fischbach, 2016; Martin, Pescosolido, Olafsdottir, & Mcleod, 2007; Pescosolido et al., 2008). We target mental illness and neurodevelopmental disorders in this paper because they are behavioral health disorders that may offer significant challenges to parents. Social distance may result from public stigma, such as not wanting children with mental health disorders to move next door or have such a child as a classmate (Ohan, Visser, Moss, & Allen, 2013). This public stigma is extended to the child’s family members (Goffman, 1963). Specifically, research shows the public negatively endorses stereotypes about adults by virtue of being parents of children with mental health and/or neurodevelopmental disorders (Corrigan, Watson, & Miller, 2006; Moses, 2014; Phelan, Bromet, & Link, 1998; Wahl & Harman, 1989). Parents of children with mental health and/or neurodevelopmental disorders have been blamed for their parental role, often leading to social distance (Corrigan et al., 2006; Eaton, Ohan, Stritzke, & Corrigan, 2016; Kinnear et al., 2016). Stereotypes that impact this blame relate to genetics (the child is stained by the bad genes of mother or father), failure to meet idealized parent standards, or not engaging the child in appropriate care.

Given these general categories, this study looks at two specific impacts of stigma: SS and vicarious stigma. Many parents experience SS; research has shown they internalize stereotypes like the ones above tagging them as “bad” parents who should be blamed for causing their child’s mental health challenges (Eaton et al., 2016; Mak & Cheung, 2012; Zisman-Ilani et al., 2013) or neurodevelopmental disorder (Mak & Cheung, 2008; Mak & Kwok, 2010). Internalized stereotypes undermine self-esteem, which subsequently leads to increased depression and diminished quality of life (QoL) (Corrigan, Bink, Schmidt, Jones, & Rusch, 2016; Mak & Cheung, 2008, 2012). Parents have also been found to suffer when they witness their child with mental health and/or neurodevelopmental disorder experience stigma (Corrigan & Miller, 2004; Robinson & Brewster, 2016; Struening et al., 2001; Wahl & Harman, 1989). Thus, parents may also experience a form of stigma that uniquely reflects the special relationship with their child: vicarious stigma. Vicarious stigmas are emotions experienced by parents when they witness their child being the object of prejudice and discrimination because of their mental health and/or neurodevelopmental disorder; for example, when the child is not invited to a peer’s birthday party or excluded from a school team due to their challenges (Eaton et al., 2016; Moses, 2014; Wahl & Harman, 1989). These kinds of experiences can lead to significant emotional response from the parents. Eaton et al. (2016) found that parents expressed feelings of sadness, guilt, frustration, and anger when vicariously experiencing their child’s stigma. Although there has been some discussion of this ‘vicarious stigma’ construct in the literature (Corrigan, 2018b; Eaton et al., 2016; Moses, 2014), we are unaware of the development or testing of a measure of vicarious stigma. One purpose of this study is to describe the development of a vicarious stigma measure comprised of hypothesized experiences representing the construct, and to provide a preliminary test of construct validity through examining its relation to SS (i.e., another measure of stigma that parents are known to experience). Based on this literature, we hypothesize that two emotions are likely to result from vicarious stigmas: sadness or anger that occurs because of their child’s exclusion due to stigma.

Experiences of different kinds of stigma by parents of children with mental health and/or neurodevelopmental disorder have demonstrated an association with various symptoms of depression such as sadness, rumination, and feelings of guilt (Eaton et al., 2016; Mickelson, 2001; Moses, 2014; Singh, 2004; Stengler-Wenzke, Trosbach, Dietrich, & Angermeyer, 2004). The impact of stigma on QoL has been documented in people with mental health challenges as well as in caregivers and family members (Corrigan, Druss, & Perlick, 2014; Corrigan & Miller, 2004). The SS perceived by an individual has been found to be associated with diminished QoL (Corrigan et al., 2010). Given these relationships, we also plan to explore the relationships between parents’ self- and vicarious stigma experiences and wellbeing. Specifically, we hypothesize that self- and vicarious (sad and angry) stigma will be positively associated with depression and inversely with QoL.

Another question addressed in this paper is stigma’s effect on disclosure and secrecy; specifically should parents disclose their child’s experience with mental health and/or neurodevelopmental disorders as a way to handle stigma experiences? Making the decision whether or not to disclose can be challenging (Corrigan & Matthews, 2003). On the one hand, more willingness to disclose has been found to lessen the negative impacts of SS on QoL and lead to personal feelings of empowerment (Corrigan et al., 2010; Yanos, Lucksted, Drapalski, Roe, & Lysaker, 2015). On the other, proponents of impression management (Bolino, Kacmar, Turnley, & Gilstrap, 2008; Neel, Neufeld, & Neuberg, 2013) believe that knowledge about a child’s symptoms and disabilities related to mental health or neurodevelopmental disorders can lead to stigma and discrimination. Hence, keeping these experiences a secret may be one way for a parent to cope with stigma that other people have towards their child (Chronister, Chou, & Liao, 2013). Secrecy coping has been defined as the degree to which people hide their mental health disorder and corresponding treatments in order to avoid the egregious effects of stigma (Link, Struening, Rahav, Phelan, & Nuttbrock, 1997; Schibalski et al., 2017; Wigand, Oexle, Staiger, Waldmann, & Rusch, 2019). Research suggests that “secrecy” can exacerbate SS, because it implies that there is something to be ashamed of (Corrigan, 2016; Corrigan & Al-Khouja, 2019). Previous literature has found the level of SS experienced by an individual can impact one’s decision to choose engaging in secrecy coping versus disclosure (Corrigan et al., 2010). In this study, we adapt the Link et al. (1997) scale of secrecy coping to assess parental views about keeping their child’s mental health or neurodevelopmental disorder secret. We then examine the relationship between secrecy coping and measures of vicarious stigma, SS, depression, and QoL. In line with hypotheses that secrecy increases self-shame (and hence SS), we expect to show that disclosure, and not secrecy coping, is positively related to indicators of wellness.

## Methods

The procedure and data for this study came from the baseline measures of a larger, ongoing project on changing family stigma of parents with children with mental health and/or neurodevelopmental disorders. Participants in this study were at least 18 years of age and a custodial parent of a child with mental health and/or neurodevelopmental disorders between the ages of 3 and 10 years. For the purposes of this study, the child’s mental health and/or neurodevelopmental disorder included ADHD, anxiety disorders, autism spectrum disorder, bipolar and related disorders, conduct disorder, depressive disorders, oppositional-defiant disorder, and posttraumatic stress disorder. We included children as young as 3-years-old because data indicate that symptoms of each of these disorders may first appear at this age or even earlier (Hopkins, Lavigne, Gouze, LeBailly, & Bryant, 2013; Lavigne, LeBailley, Hopkins, Gouze, & Binns, 2009). Children with diagnoses of intellectual disability were excluded because a previous study has indicated that parents’ stigma might be different for this group (Mak & Cheung, 2008). Parents of children with autism spectrum disorder may have similar, parent-blaming experiences of stigma parents of children with mental health disorders experience (Mak & Kwok, 2010), and this may also be true for parents of children with ADHD relative to other mental health disorders (Eaton et al., 2016), so they were included in this study as well.

Interested participants were contacted through National Alliance on Mental Illness (NAMI) and other advocacy groups across Wisconsin through flyers sent vis existing listservs, on e-bulletin boards, or in actual NAMI offices. Interested participants contacted a phone interviewer for more information and were assessed for eligibility. If all inclusion criteria were met, participants were given the option to complete consent form and measures online (n = 40) or to make an appointment in order to fill out a consent form and complete paper-and-pencil measures in person (n = 10). No differences were found in online versus paper-and-pencil measures so data were collapsed across groups for this study. Research participants were fully informed to the study and provided written consent before beginning. Parent participants received $15 for completing the 20-min survey. All aspects of the study were approved by the Institutional Review Board at the Illinois Institute of Technology. This work was supported by the Rogers Memorial Hospital Foundation. Authors declare no known conflicts of interests and are fully responsible for the content and writing of this article.

### Measures

Research participants completed measures of demographics, vicarious stigma, SS, secrecy coping, depression, and QoL. Internal consistency for each measure was calculated for the sample in this study and reported below. Demographic factors were selected that correspond with existing stigma research literature and reflected parent descriptors (Parcesepe & Cabassa, 2013) and included year of birth, gender, ethnicity, race, marital status, employment status, yearly family income, highest level of achieved education, and custodial relationship to their child (e.g., biological parent, step-parent, adoptive parent). We also collected demographics that were specific to questions of parenting children with mental health and/or neurodevelopmental disorders. These included items about the child included year of birth, gender, primary diagnoses, approximate year of diagnosis, professional who provided the diagnosis, and type of treatments/supports the child was currently receiving.

We developed the Vicarious Stigma Scale (VSS) for this study using previously described methods for developing measures of stigma (Corrigan, 2018a). We constructed vignette-based items representing hypothesized experience that yield vicarious stigma to which respondents provided responses on Likert scales. Authors by consensus identified seven situations experienced by a child with mental health and/or neurodevelopmental disorders. For example, my child doesn’t get chosen to be part of a sport’s team or does not get invited to a party because of his/her mental health or neurodevelopmental disorder. Items then represented the emotional reaction of stigmas, specifically in terms of sadness and anger. Participants were instructed to respond to each situation with two 10-point scales: “How much does this make you feel sad…?” or “How much does this make you feel angry…?” (10 = very much). Scores were summed to yield individual vicarious stigma sad and anger scores. Higher scores reflected more vicarious stigma. Internal consistencies of participant responses in our study were satisfactory for each subscale (VSS-anger α = 0.79 and VSS-sadness, α = 0.78).

Remaining measures were then selected to represent key constructs to which vicarious stigma was compared: SS, secrecy, depression and QoL. Self-stigma was assessed by using the Parents’ Self-Stigma Scale (PSSS; Eaton, Ohan, Stritzke, & Corrigan, 2016, 2019). This 11-item, self-report measure asks participants to rate items on five-point agreement scales (5 = almost all the time). Sample items include “I feel guilty that my child has his/her problem.” and “I’m not a good enough parent.” Total score is computed by calculating the sum of items. Higher scores represent higher levels of SS. The PSSS has demonstrated sufficient reliability (Eaton et al., 2019) for this study α = 0.83.

Secrecy coping was measured using an adapted and expanded version of the 5-item Secrecy Coping Scale (Link et al., 1997). This measure was adapted by rewording phrases about an adult’s mental illness to reflect the experiences of parents of children with mental health and/or neurodevelopmental disorders (e.g., “For me to be successful at work, it is best to hide my diagnosis or problems.” was changed to “For my child to be successful at school, it is best to hide his/her diagnosis or problems.”). In addition, four items were added to address a limit of the original scale; namely parents choosing secrecy as a coping mechanism to benefit themselves, not their child (e.g., “I try to hide my parenting struggles from others.”). Participants were instructed to rate each item on a 6-point Likert scale (6 = strongly disagree). Half the items were reverse scored. Total scores were computed by calculating the sum of items. Lower scores represent greater levels of secrecy coping, and less promotion of disclosure. Evidence supports good reliability of the original scale (α = 0.72) (Link et al., 1997); alpha was similarly satisfactory for our data (α = 0.87).

Depression was measured using the Centre for Epidemiological Studies Short Depression Scale (CESD-R-10; Andersen, Malmgren, Carter, & Patrick, 1994). This 10-item, self-report measure asks participants to respond to items (e.g., “I felt depressed”) using a rating scale (rarely or none of the times (less than 1 day) to all of the time (5–7 days). The total score was computed by calculating the sum of items. Higher scores represent greater levels of depression. The CESD-R-10 has demonstrated sufficient reliability and validity (α = 0.89) (Andersen et al., 1994; Björgvinsson, Kertz, Bigda-Peyton, McCoy, & Aderka, 2013). Alpha was also in satisfactory range for our research participants (α = 0.86).

QoL for parents was assessed using the Lehman Quality of Life Interview (QOLI; Lehman, 1988). The 6-item, self-report measure asks participants to rate each item using a 7-point Likert scale (1 = terrible to 7 = delighted) by asking, “how do you feel about…?” The total score was computed by calculating the sum of items. High scores represent higher levels of QoL. Evidence supports good reliability of the QOLI (α = 0.91) (Lehman, 1988; McNary, Lehman, & O’Grady, 1997). Alpha for our respondents was good (α = 0.86).

### Statistical Analyses

Missing data were identified and imputations conducted where appropriate. Continuous variables were assessed to meet the assumptions of linear regression, including variable distributions for normality using skew and kurtosis. Although the factor structure of the VSS is best tested using confirmatory factor analyses, we were underpowered to do so. Therefore, we sought to provide preliminarily factor structure evidence using exploratory factor analysis (EFA). We did a control analysis to determine whether demographics were significantly associated with conceptual variables using Pearson Product Moment Correlations. Subsequent analyses would have been done as partial correlations if any consistent association weas found between demographic and conceptual variable. Pearson product-moment correlations were then used to estimate associations between SS, vicarious stigmas, secrecy coping, and the outcome variables of depression and QoL. Specifically, one set of analyses examined correlations between target constructs of vicarious and SS and its impact on depression. A second set of analyses examined the concurrent impact of secrecy on these variables. We used Bonferroni corrections to control for type I error. Subsequent multiple linear regressions estimated the independent effects of SS, vicarious-stigma, and secrecy coping on depression and QoL. All analyses were conducted using SPSS v. 23 (IBM Corp., 2015).

## Results

Demographics for the 50 participants are summarized in Table 1. The sample was 94% female and, on average, 36.8 years old. Participants were largely white, married and at least partly employed. Eighty percent of participants were biological parents. Two-thirds of children were male and 8.0 years old on average. The most frequent primary diagnoses reported were ADHD and autism spectrum disorder. On average, they had been diagnosed for 2.61 years. Pearson product moment correlations were determined between selected demographic variables and the measures of stigma and harm as part of the control analysis. Only two of the thirty correlation coefficients were significant, suggesting partial correlations were unnecessary. Older parents endorsed secrecy coping less (*r* = 0.28, *p* < 0.05) and parents with male children endorsed Vicarious Stigma-Anger (VSA) more (*r* = 0.29, *p* < 0.05).

**Table 1:** Demographics of research participants (N = 50)

Variable	% (N) or M (SD)
Age	36.8 (6.8)
Gender	
Female	94% (47)
Male	6% (3)
Race	
African American, Black	12% (6)
Asian	2% (1)
White, Caucasian	86% (43)
More than one race	2% (1)
Ethnicity	
Hispanic/Latino	4% (2)
Not Hispanic/Latino	94% (47)
Marital status	
Single	26% (13)
Married	66% (33)
Divorced	8% (4)
Employment status	
Not employed outside of house	24% (12)
Full-time	60% (30)
Part-time	16% (8)
Yearly family income	
$0–$25,000	8% (4)
$25,001–$49,999	38% (19)
$50,000–$74,999	30% (15)
$100,000–$149,000	14% (7)
Educational attainment	
Some high school	4% (2)
High school	10% (5)
Some college	26% (13)
College degree	28% (14)
Some graduate school	12% (6)
Graduate degree	16% (8)
Relationship to child	
Biological parent	80% (40)
Step-parent	4% (2)
Foster parent	2% (1)
Adoptive parent	14% (7)
Child age	8.02 (2.3)
Child gender	
Female	32% (16)
Male	68% (34)
Child primary diagnosis	
Attention-deficit/hyperactivity disorder	22% (11)
Oppositional-defiant disorder	4% (2)
Conduct disorder	2% (1)
Post-traumatic stress disorder	6% (3)
Bipolar disorder	4% (2)
Depression	2% (1)
Anxiety	8% (4)
Autism spectrum disorder	26% (13)
Other	12% (6)
Years diagnosed	2.61 (2.05)
Who diagnosed?	
Pediatrician, GP, or other medical doctor (MD)	18% (9)
Psychologist	32% (16)
Psychiatrist	20% (10)
Other	14% (7)
Treatment/support type	
Medication	56% (28)
Parenting classes	18% (9)
Family therapy	26% (13)
Individual child therapy	46% (23)
Other	32% (16)

A total of 35 (70%) participants had at least one missing data point; 12.2% of scale items were missing. Results for Little’s MCAR test were not significant, indicating data was missing at random. Missing data were managed by implementing a stochastic regression imputation method (van Buuren & Groothuis-Oudshoorn, 2011; Sarkar, 2008), since it is a less biased method of imputation than other methods (Brockmeier, Kromrey, & Hines, 1998). Four participants inadvertently were not provided with the depression and QoL scales, so the sample size for these analyses was 46, and data from these cases were not imputed for these two scales. The range of absolute values of test score skewness ranged from 0.29 to 0.74 which indicated little to moderate skew. The range of absolute values of test score kurtosis ranged from 0.11 to 0.96 which is well within acceptable limits. Hence, values were not transformed to yield a normal distribution.

Findings from an EFA (principle component analysis with varimax rotation) for the Vicarious Stigma Scale are summarized in Table 2, which provides factor loadings with eigenvalue set to 2.0. Values for Bartlett’s test of sphericity [*χ*^2(91)^ = 300.46, *p* < 0.001] and the Kaiser–Mayer–Olkin measure of sampling adequacy (KMO = 0.644) were satisfactory (Yong & Pearce, 2013). Review of the factor loadings show six of the seven items meet criterion (0.55) for factor 1, the VSA factor. The item that was excluded was, “relatives exclude my child from family functions because of his/her mental health problems.” Six of the seven items met criterion for factor 2, the VSS factor. The item that was excluded was, “my child is excluded from religious functions because of his/her mental health problems.” VSA and VSS were found to be significantly correlated (*r* = 0.31, *p* = 0.01).

**Table 2:** Factor loadings for exploratory factor analyses with varimax rotation of the vicarious stigma scale (N = 50)

Item	Component	
	(1) Anger	(2) Sad
How much does each of the following situations make you feel **SAD**?		
My child doesn’t get invited to a party because of his/her mental health problems.	0.07	**0.63**
Someone makes fun of my child because of his/her mental health problems.	0.03	**0.76**
Other children do not play with my child because of his/her mental health problems.	0.09	**0.65**
My child doesn’t get chosen to be part of a sport’s team because of his/her mental health problems.	−0.13	**0.73**
Relatives exclude my child from family functions because of his/her mental health problems.	0.12	**0.65**
A teacher overlooks my child at school because of his/her mental health problems.	0.52	**0.57**
My child is excluded from religious functions because of his/her mental health problems.	0.27	0.49
How much does each of following situations make you feel **ANGRY**?		
My child doesn’t get invited to a party because of his/her mental health problems.	**0.55**	−0.08
Someone makes fun of my child because of his/her mental health problems.	**0.77**	0.12
Other children do not play with my child because of his/her mental health problems.	**0.83**	0.05
My child doesn’t get chosen to be part of a sport’s team because of his/her mental health problems.	**0.66**	0.07
Relatives exclude my child from family functions because of his/her mental health problems.	0.40	0.45
A teacher overlooks my child at school because of his/her mental health problems.	**0.67**	0.33
My child is excluded from religious functions because of his/her mental health problems.	**0.60**	0.11

*Note*. Factor loadings >0.55 are in boldface

Pearson Product Moment Correlation Coefficients, means, and standard deviations of study measures are summarized in Table 3. Bonferroni correction set the p value at 0.05/15 = 0.003; there were 15 correlation coefficients in the table. Noticeably, VSA was not significantly associated with SS or the two outcome indicators. VSS, on the other hand, was significantly associated with depression and QoL though this did not meet Bonferroni criterion. Parents with less VSS were likely to report greater QoL and less depression. SS showed a similar set of significant relationships; parents with greater SS reported greater depression and diminished QoL. The association between SS and depression met the Bonferroni Criterion. A significant relationship was found between VSS and SS thought it did not meet the Bonferroni criterion. Participants with greater SS were more likely to report sadness after their child experienced stigma situations.

**Table 3:** Pearson product moment correlations among indices of vicarious stigma, self-stigma, secrecy coping, depression, and QoL

Measure	VSS	VSA	SS	SC	Dep	QoL
VSS						
VSA	0.31**					
SS	0.25*	−0.08				
SC	0.04	−0.08	−0.30*			
Dep	0.31*	0.05	0.49***	−0.44***		
QoL	−0.31*	0.14	−0.38**	0.41***	−0.60***	
M	6.46	6.25	2.39	4.47	2.23	4.60
SD	2.47	2.60	0.71	1.02	0.67	0.94

*Note*. These values represent 1-tailed t-tests. VSS = vicarious stigma-sad; VSA = vicarious stigma-anger; SS = self-stigma; SC = Secrecy coping; Dep = depression; QoL = quality of life

*p < 0.05, **p < 0.01, ***p < 0.005

Table 3 also summarizes relationships between secrecy coping with the stigma and outcome measures. The secrecy coping index was not found to be associated with either of the vicarious stigma factors but was negatively associated with SS though not meeting Bonferroni criterion. The latter finding suggests parents who are less secretive, or are more willing to disclose, report less SS. Specifically, parents who admitted to using secrecy to cope with stigma showed greater depression and diminished QoL.

Table 4 summarizes multiple regression analyses that examined the independence of stigma and secrecy relationships with depression and QoL. The first equation with depression as the dependent variable yield *R* = 0.62, *F*(3,42) = 8.81, *p* < 0.001. Betas for two of the three independent variables were significant, suggesting secrecy coping and SS each accounted for independent variance in depression. The beta for sadness due to vicarious stigma was a nonsignificant trend. The second equation with QoL had an *R* = 0.55, *F*(3,42) = 6.18, *p* = 0.001. Beta values for two of the three variables were significant: sadness due to vicarious stigma and secrecy coping. Hence, these two variables independently explained variance in QoL.

**Table 4:** Multiple regressions examining the independence of impact of vicarious stigma, self-stigma, and secrecy coping on depression and QoL

Dependent variable: Depression	R = 0.62	
Independent variable	Beta	t-test (p)
Vicarious stigma-sad	0.24	1.88 (0.067)
Self-stigma	0.33	2.48 (0.017)
Secrecy coping	−0.35	−2.74 (0.009)

Dependent variable: Quality of Life	R = 0.55	
Independent variable	Beta	t-test (p)
Vicarious stigma-sad	−0.27	−2.02 (0.050)
Self-stigma	−0.21	−1.50 (0.141)
Secrecy coping	0.35	2.58 (0.013)

## Discussion

Parents of young children with mental health and/or neurodevelopmental disorders may be harmed by stigma themselves. Two types of parents’ stigma were examined here: (a) SS, a loss of self-esteem when parents internalize stereotypes about themselves as bad (e.g., “I’m not a good enough parent”), and (b) vicarious stigma, reacting with sadness or anger when parents observe their child being discriminated against. Findings from this study supported some of our hypotheses about the harmful effects of both kinds of stigma. Parents with greater SS were found to show more depression and lower QoL. These findings parallel results from earlier research; namely, parents suffer diminished self-esteem and self-efficacy when they internalize negative stereotypes about themselves as parents (Eaton et al., 2019; Mak & Cheung, 2008, 2012; Mak & Kwok, 2010). It seems that concerns about the impact of SS for people with mental health and/or neurodevelopmental disorders are extended to their close associates. More unique were our findings of a relatively newer construct; that is, vicarious stigma. We hypothesized and tested two emotional consequences: sadness and anger. Parents who reported greater sadness due to vicarious stigma had greater depression and lower QoL. This effect seems unlike others identified in the stigma literature. Here, harm occurs secondarily to the direct effects of stigma toward one’s child. Interestingly, anger due to vicarious stigma was not found to be significantly associated with either depression or QoL. This absence of findings fails to support the construct validity of anger as vicarious stigma.

We also examined associations between wellbeing and secrecy coping. On one hand, coping through secrets may have beneficial effects, hiding the child’s mental health and/or neurodevelopmental disorder is a form of positive impression management, so that the child escapes stigma’s harm. Impression management describes efforts to create, maintain, protect, or otherwise alter an image held by a others (Bolino et al., 2008). On the other hand, not keeping it a secret—actually disclosing—may undermine self-shame and create avenues of support, thereby positively impacting depression and QoL. Findings from our study support the latter. Secrecy coping was associated with depression; parents who were more inclined to secrecy reported higher depression. Similarly, those who were more likely to disclose reported better QoL. These findings advance growing evidence about the value of disclosure for managing SS (Corrigan, Kosyluk, & Rusch, 2013). In previous research, disclosure seems to have value when people decide for themselves to share their own experience. Here, value is observed when people decide to share others’ experiences, in this case, their child’s.

Secrecy here was framed in terms of disclosure. Unfortunately, the construct presented here may have been over-simplified. It was framed as whether parents should disclose as a way to handle stigma. This kind of yes-no categorical question limits the nuance more likely in parent decision making. More accurate might be examining when and how should parents disclose.

We conducted multiple regression analyses to determine overlap among the independent variables associated with depression and QoL. Anger due to vicarious stigma was excluded because it was not significantly associated with either outcome in the bivariate correlations. Results of the two regressions suggest some overlap between sadness due to vicarious stigma and SS. In one case, SS and not vicarious stigma were significantly associated with depression, while in the other, vicarious stigma and not SS was significantly related to QoL. This corresponded with a significant trend in the bivariate relationship. This finding suggests some overlap in the experience of self- and vicarious stigma, at least as assessed in current measures. Future research needs to more fully explore this relationship. Interestingly, the EFA yielded a surprising level of differentiation between the two domains. Of course, this may have resulted as an artifact of the second step rotation in EFA. Still future research needs to better explain this level of differentiation.

There are limitations to this study that need to be considered in future work. Among other things, research participants did not respond to actual experiences with their child but rather to hypothesized situations. This task may have diminished reliability and validity of the task. It is also possible that anger is not related to wellbeing for parents; for example, Corrigan and Watson (2002) have discussed the feeling of ‘righteous indignation’ as a coping style for dealing with stigma. It could be that the anger experienced as what we assumed to be ‘vicarious stigma’ is part of a coping style that rejects the stigma, and thus the parent does not internalize the damaging stigmatic message. Both vicarious stigma measures were developed for this study and hence need additional psychometric work. Future research might wish to examine the content validity of item candidates using community-based participatory research (CBPR); Corrigan (2020) argued CBPR is essential for yielding stereotypes that in fact represent the perspective of those harmed by stigma. Alternatively, as stated above, anger may not be a valid construct for understanding one emotional response to vicarious stigma. Research on vicarious stigma also needs to explain its potency; what is the depth of harm experienced by a parent when observing their child’s harm due to stigma?

The Secrecy Coping Scale was adapted from another instrument for this study; hence, reliability and validity of its items need to be further examined as well. Additional research needs to examine how characteristics of parents and their children impact findings. For example, larger samples may facilitate examining the impact of the parent’s age, gender, ethnicity, and partner status (e.g., single parent). Similarly, future research could include descriptors of the child such as diagnosis, age, and treatment history, which might prove to be moderators or mediators of findings. Examination of mediator effects, for example, could determine whether the relationship between SS and depression goes through the child’s age or gender. Subsequent research should also examine moderating or mediating effects of secrecy coping on the relationship between stigma outcomes. This study was underpowered to do so. Future research should determine whether secrecy coping mutes the effects of stigma on depression and QoL. Additional research should examine types of stigma, secrecy coping, depression, and QoL longitudinally experienced by parents of children with mental health and/or neurodevelopmental disorders in order to disentangle these relationships.

The study was limited by a cross sectional data collection. Better inferences about direction of effects could be determined in future research through a cross-panel design. Moreover, research needs to examine generalizability of findings. Note that participants form this study were recruited from Wisconsin; future research needs to determine how these findings play out in other geographic sections of the US, as well as internationally.

Findings from this study have implications for ongoing efforts to understand and diminish the stigma of mental illness. Vicarious stigma is an additional construct for mapping the harmful effects of prejudice and discrimination (Brohan et al., 2010), in this case, as another example of associative stigma (Goffman, 1963). Future research should examine the dynamics of self- and vicarious stigma with the parent and through relationships with the child. Vicarious injury is likely to be exacerbated by the nature of the relationship, especially parental responsibility for their young child. In particular, future research should examine whether vicarious and self-sigma vary between mental health and neurodevelopmental disorders.

This kind of knowledge will inform anti-stigma programs. Once again, disclosure seems to have benefits for decreasing stigma’s harm. Research needs to better understand what it means to disclose someone else’s stigmatizing experiences and its impact on both parties. Honest, Open, Proud (HOP) is one program that has been developed to diminish stigma by helping people decide whether and how to disclose their experiences with mental illness (Mulfinger et al., 2018). CBPR might be done to determine how to adapt HOP for parents. How might the costs and benefits of disclosure vary by setting; e.g., the child’s school, the parent’s workplace, or the extended family? What story should the parent state? Unlike HOP for adults, part of the disclosure process here is sharing another person’s mental illness: their child’s. HOP adaptation needs to include the child in the adaptation process, especially as children age. Young children (under six) may have less strong opinions about this compared to teenagers.
